# CFAP300 Loss-of-Function Mutations with Primary Ciliary Dyskinesia: Evidence from Ex Vivo and ALI Cultures

**DOI:** 10.3390/ijms26157655

**Published:** 2025-08-07

**Authors:** Anna G. Demchenko, Tatiana A. Kyian, Elena I. Kondratyeva, Elizaveta E. Bragina, Oksana P. Ryzhkova, Roman V. Veiko, Aleksandra G. Nazarova, Vyacheslav B. Chernykh, Svetlana A. Smirnikhina, Sergey I. Kutsev

**Affiliations:** 1 Laboratory of Genome Editing, Research Centre for Medical Genetics, 115522 Moscow, Russia; 2 Scientific and Clinical Department of Cystic Fibrosis, Research Centre for Medical Genetics, 115522 Moscow, Russia; 3 Laboratory of Genetics of Reproductive Disorders, Research Centre for Medical Genetics, 115522 Moscow, Russia; 4 A.N. Belozersky Institute of Physico-Chemical Biology Lomonosov Moscow State University, 119234 Moscow, Russia; 5 Laboratory of Molecular Genetic Diagnosis 1, Research Centre for Medical Genetics, 115522 Moscow, Russia; 6 Laboratory of Molecular Biology, Research Centre for Medical Genetics, 115522 Moscow, Russia; 7 Administration, Research Centre for Medical Genetics, 115522 Moscow, Russia

**Keywords:** primary ciliary dyskinesia, CFAP300, air–liquid interface culture, transmission electron microscopy, immunofluorescence

## Abstract

Primary ciliary dyskinesia (PCD) is a genetically heterogeneous disorder characterized by impaired mucociliary clearance due to defects in motile cilia. This study investigates the impact of loss-of-function mutations in the *CFAP300* gene on the ciliary structure and function in three PCD patients. Using a multimodal approach, we integrated molecular genetic testing, transmission electron microscopy, the high-speed video microscopy assay and immunofluorescence staining to analyze ciliary motility and protein expression in both ex vivo and in vitro-obtained ciliary cells. Our results revealed that the pathogenic variant c.198_200delinsCC (p.Phe67ProfsTer10) in *CFAP300* led to the absence of the functional CFAP300 protein, the complete loss of outer and inner dynein arms and immotile cilia. Air–liquid interface (ALI)-cultured cells from patients exhibited no ciliary beating, contrasting with healthy controls. Immunostaining confirmed the absence of CFAP300 in patient-derived cilia, underscoring its critical role in dynein arm assembly. These findings highlight the diagnostic utility of ALI cultures combined with functional and protein analyses for PCD, offering a clinically actionable framework that can be readily incorporated into standard diagnostic workflows.

## 1. Introduction

Primary ciliary dyskinesia (PCD) is a genetically heterogeneous disorder caused by structural and functional defects in motile cilia, leading to impaired mucociliary clearance. This condition manifests clinically as chronic respiratory infections, bronchiectasis, male infertility, and, in some cases, situs inversus [1,2]. PCD is primarily inherited in an autosomal recessive manner, though rare autosomal dominant and X-linked forms have been reported [3]. To date, over 60 genes encoding ciliary structural or assembly proteins have been implicated in PCD, contributing to its significant genetic diversity [4]. Mutations in these genes disrupt axonemal integrity, often resulting in the absence of outer (ODA) and/or inner dynein arms (IDAs), which are essential for proper ciliary motility. Among PCD-associated genes, the cilia and flagella-associated protein (CFAP) family has garnered particular interest due to its direct role in axonemal structure and function. Mutations in *CFAP300* (also known as *C11orf7*) are inherited in an autosomal recessive manner and directly associated with the loss of ciliary function, as the dynein arms are critical for generating the force required for ciliary beating. Previously, Schultz et al. identified a loss-of-function (LoF) mutation in *CFAP300* in Finnish patients with PCD in the homozygous state, resulting in the immotility of airway cilia and an absence of dynein arms [5]. Other CFAPs, such as *CFAP43*, *CFAP44* and *CFAP74*, have also been associated with PCD, although often with milder defects of ciliary function [6,7].

The diagnosis of PCD remains challenging due to the overlap of clinical features with other respiratory diseases, extensive genetic heterogeneity and the variability in ciliary ultrastructural defects. As a result, a comprehensive diagnostic approach that combines clinical evaluation with specialized laboratory techniques is essential for accurate patient identification [8,9,10]. European guidelines recommend a combination of specialist tests: the measurement of nasal nitric oxide (nNO), high-speed video microscopy analysis to assess ciliary beat frequency and pattern (CBF and CBP, respectively), transmission electron microscopy (TEM) of ciliary ultrastructure, and genetic testing [11]. However, there is no «gold standard» for diagnosing PCD. TEM has limited sensitivity, as 20–30% of PCD cases exhibit a normal ciliary ultrastructure, thus rendering the TEM method unsuitable for universal diagnosis [12]. Additionally, molecular genetic diagnosis is complicated by the lack of clear genotype–phenotype correlations and the large number of disease-causing genes. Therefore, the functional assessment of cilia (CBF and CBP) is a significant diagnostic method [11]. Ciliary function can be evaluated ex vivo (using freshly obtained nasal epithelial samples) or in vitro (via cultured ciliated cells) [13,14]. Ex vivo ciliary samples frequently exhibit secondary ciliary dyskinesia (SCD), which may be the result of various external factors such as infections, toxic effects, and other diseases that may temporarily or permanently impair cilia function [15,16]. Therefore, in vitro ciliogenesis offers the advantage of excluding signs of SCD, as well as allowing the controlled conditions required to carry out a functional assessment of cilia. Furthermore, it is possible to keep cilia in culture for a long time to conduct repeated tests if necessary. There are several methods of in vitro ciliogenesis that have been described in the literature. The most common of these is the air–liquid interface (ALI) culture of nasal epithelium [14,17,18]. Other methods include the formation of apical spheroids [19,20] and organoids [21,22], in which the rate of rotation of spheroids/organoids is evaluated. Studies have demonstrated that in cilia grown in vitro using ALI culture, both structure and function can be analyzed, aiding in the diagnosis of PCD [16,17,18,23]. For instance, in the study of Coles et al., ALI cultures demonstrated high diagnostic utility, achieving 82.9% ciliogenesis success and resolving 63.9% of inconclusive ex vivo cases [14]. Similarly, Kurokawa et al. used ALI-cultured cells to detect abnormal ciliary beating in patients with mutations in *DNAH11*, despite their normal TEM results [23]. The immunofluorescence assay serves as a complementary tool in the diagnostic workflow, enabling the visualization of axonemal protein localization and the identification of their absence or mislocalization in ciliary epithelium samples. The absence or mislocalization of key proteins (e.g., CFAP family members) provides direct evidence of molecular defects [5,8]. This approach, when combined with molecular genetic testing and functional assays, enhances the sensitivity and specificity of PCD diagnosis [9].

In this study, we employed a multimodal approach, integrating molecular genetic testing, functional ciliary analysis (using both freshly harvested and ALI-cultured ciliary cells), and immunostaining, to investigate ciliary function and protein expression in patients with *CFAP300* mutations, comparing them to healthy controls.

## 2. Results

### 2.1. Clinical Characteristics of PCD Patients

All three patients exhibited severe disease manifestations with impaired lung function. Patients underwent structured interviews and clinical evaluations using the PICADAR prediction tool. Patients achieving a PICADAR score ≥ 10 have >90% probability of having PCD confirmed through definitive diagnostic testing. A score ≥ 5 shows a clinically significant predictive value, indicating >11% probability of ultimately receiving a PCD diagnosis. The data of the study participants are presented in Table 1. Additionally, the male patient (PCD2) was diagnosed with infertility, while the 35-year-old female patient (PCD3) presented with a history of failed conception and is currently undergoing preparation for IVF (in vitro fertilization).

### 2.2. Characterization of Nasal Epithelium Samples Ex Vivo

**Figure 1:**
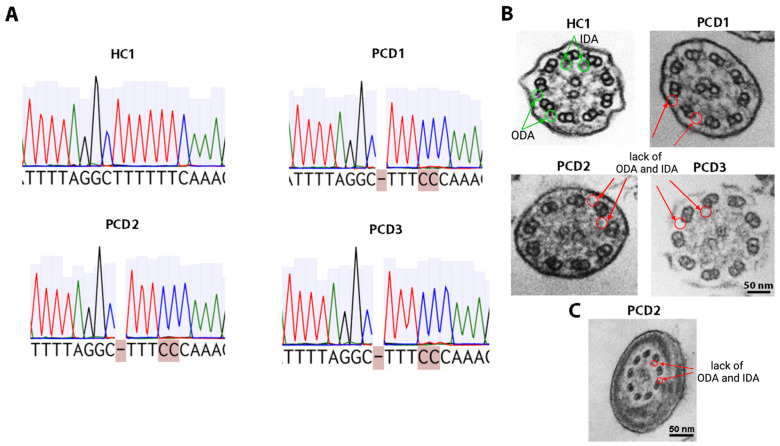
Molecular and ultrastructural analysis in PCD patients (PCD1, PCD2, PCD3) and a healthy donor (HC1). (**A**) Sanger sequencing results for the *CFAP3000* gene mutation region. (**B**) Transmission electron microscopy of a cilia. Arrows and circles indicate the presence of ODA and IDA in a healthy donor and their absence in three PCD patients. Scale bar 50 nm. (**C**) Transmission electron microscopy of a spermatozoon from a PCD2 patient. Arrows and circles indicate the absence of ODA and IDA. Scale bar 50 nm.

Nasal epithelial samples were obtained from three patients with PCD and three healthy donors. The whole-exome sequencing and Sanger sequencing results from all patients revealed the pathogenic variant c.198_200delinsCC (p.Phe67ProfsTer10) at chr11:102058886-102058888 of the *CFAP300* gene in the homo/hemizygous (Figure 1A). The identified pathogenic variant, previously described as pathogenic in the homozygous state in PCD 38 (OMIM: 618058.0005) [5,8], leads to the loss of functional CFAP300 protein due to a frameshift-induced premature stop codon, resulting in nonsense-mediated mRNA decay or the synthesis of a shortened nonfunctional protein.

The TEM of cilia results revealed the absence of ODA and IDA in all PCD patients (Figure 1B). TEM analysis of the ciliated epithelium sample for HC1 was performed, showing a normal cilia structure. Andrological examination with standard spermiological examination (spermogram analysis) was performed on a PCD2 male. The patient was found to have total asthenoteratozoospermia (the number of motile and morphologically normal spermatozoa–0). Electron microscopy of spermatozoa revealed an abnormal axonemal structure in the flagellum, specifically the complete absence of dynein arms, which is consistent with the TEM findings in this patient’s nasal epithelium (Figure 1C).

The ex vivo video microscopy of native nasal epithelium samples from PCD1 and PCD3 revealed immotile cilia with residual CBF values of 1.25 Hz (0.3–1.9) and 0.9 Hz (0.5–1.0), respectively (Table 2), confirming a LoF protein defect. In contrast, healthy donor samples showed regular CBP with significantly higher CBF: HC1: 6 (5.5–6.3); HC2: 5.6 (4.4–6); HC3: 6.1 (4.9–6.6). CBF differed significantly (*p* < 0.0001, nested *t*-test) between PCD patients (median 1.1 Hz) and healthy donors (median 5.8 Hz).

### 2.3. Characterization of Ciliated Epithelium Samples In Vitro

**Figure 2:**
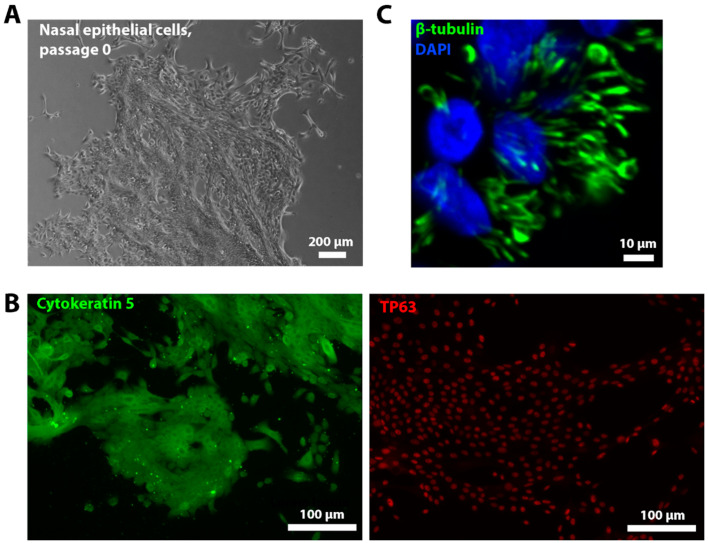
Characterization of nasal epithelial cells and ALI cultures. (**A**) A phase-contrast image of the cells isolated during brush biopsy at the zero passage. Scale bar 200 μm. (**B**) Representative fluorescence images of nasal epithelial cells stained for epithelial cell markers (cytokeratin 5 and TP63). Scale bar 100 μm. (**C**) Representative fluorescence images of cilia (β-tubulin) in ALI culture at 24 d of ciliogenesis in vitro. Nuclei were stained with DAPI (blue). Scale bar 10 μm.

Nasal epithelial cell cultures were obtained from three patients with PCD and three healthy donors. Figure 2A demonstrates a phase-contrast image of the cells at zero passage. Figure S1 presents phase-contrast images of all six cell cultures at passage 0. To confirm the epithelial origin of the derived cells, immunofluorescence staining was performed for two major markers of basal epithelial cells, tumor protein p63 (TP63) and cytokeratin 5. Both markers positively stained the obtained cells (Figure 2B). After obtaining cells, the cell lines were biobanked and differentiated into ciliated cells by ALI culturing. Positive immunofluorescence staining for the ciliated cell marker β-tubulin confirmed the successful generation of a ciliated epithelium in vitro (Figure 2C). On 24 d of differentiation, video microscopy analysis of ALI cultures (*n* = 3 for each donor) was performed with the evaluation of CBF and CBP (Table 3). The quantitative analysis revealed complete ciliary immotility in PCD-derived ALI cultures, with only background-level CBF detected (median 1.5 Hz). CBF in ALI cultures differed significantly between PCD patients and healthy donors (*p* = 0.0008, nested *t*-test). Coordinated ciliary beating was observed in ALI cultures derived from healthy donors (median 7.7 Hz). In our study, the CBF of in vitro-obtained ciliated epithelium samples from PCD patients was significantly higher than that of native tissue samples (*p* = 0.0118). In contrast, CBF in healthy donors showed no significant difference between the in vitro-obtained ciliated epithelium and ex vivo samples (*p* = 0.1269). The slight CBF difference between ex vivo and in vitro ciliary samples in PCD patients may result from technical artifacts during brush biopsy collection (e.g., tissue damage), mucus contamination, or underlying inflammation in native specimens.

**Figure 3:**
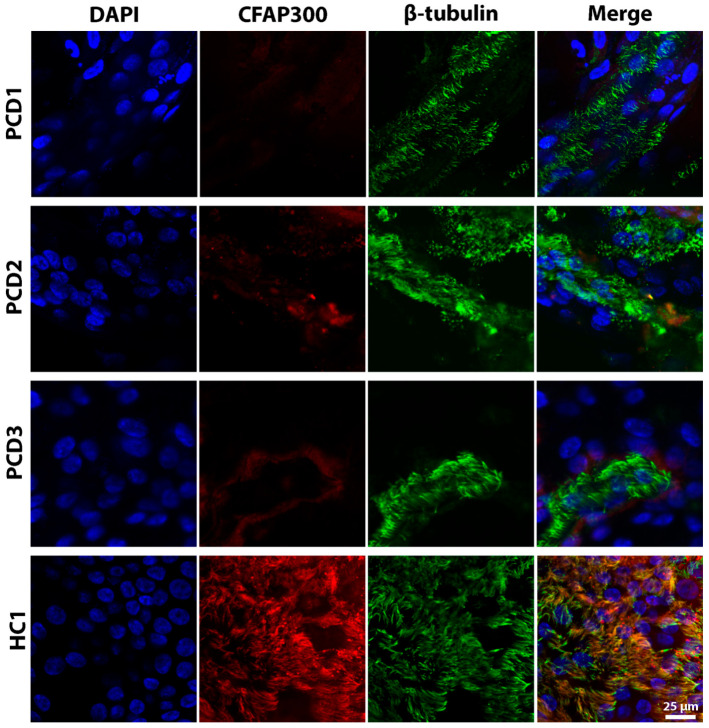
Fluorescence images of cilia in ALI cultures of patients with loss-of-function mutations in the *CFAP300* gene and a healthy donor (HC1) stained for CFAP300 and β-tubulin. Nuclei were stained with DAPI (blue). Scale bar 25 μm.

ALI cultures at 24 d of differentiation, derived from PCD patients and healthy donors, were stained for CFAP300 (Figure 3). In the ALI cultures of healthy donors, CFAP300 localized to ciliated cells (co-stained with β-tubulin), whereas ALI cultures from patients with LoF mutations in CFAP300 showed a complete absence of CFAP300 expression in ciliated cells. The colocalization analysis revealed significant differences between the groups. The Manders’ coefficient averaged 0.44 ± 0.16 in healthy subjects, significantly higher than the 0.20 ± 0.18 observed in PCD patients (*p* = 0.022). Similarly, the RWC in healthy donors averaged 0.35 ± 0.13, significantly exceeding the 0.14 ± 0.12 observed in PCD patients (*p* = 0.008). These findings suggest that CFAP300 staining could serve as a diagnostic tool to confirm molecular defects in patients suspected of PCD. However, additional studies with larger patient and donor cohorts are required to validate the universality and clinical applicability of this marker.

## 3. Discussion

Our study provides compelling evidence that LoF mutations in the CFAP300 gene lead to severe ciliary dysfunction in patients with PCD. We investigated adult patients (aged 23–35 years) with advanced, multisystem manifestations of PCD. All three cases exhibited severe pulmonary involvement, including progressive bronchiectasis, chronic Pseudomonas aeruginosa infection, and conductive hearing loss, with two fulfilling the diagnostic criteria for Kartagener syndrome. In previous studies, LoF mutations in *CFAP300* were predominantly identified in pediatric or adolescent patients, who typically presented with milder, early-stage symptoms due to limited disease progression at evaluation [5,24,25]. PCD is known to cause male infertility, because sperm flagella are special motile cilia that can also exhibit defective motility [6,26,27,28]. In our study, a male patient with the pathogenic variant c.198_200delinsCC in *CFAP300* was found to have total asthenoteratozoospermia resulting in a total absence of dynein arms, which is consistent with the results of Hoben et al., where asthenoteratozoospermia was detected in patients with an LoF mutation in *CFAP300* [8]. Our findings suggest that disease progression in CFAP300-associated PCD is age-dependent, which underscores the critical need for early genetic and functional diagnostics, as well as the utility of multimodal monitoring in older PCD patients to anticipate and manage advanced complications.

Through a combination of molecular genetic analysis, ultrastructural and functional ciliary assessment and immunostaining, we demonstrated that the pathogenic variant c.198_200delinsCC (p.Phe67ProfsTer10) in *CFAP300* results in the absence of the functional CFAP300 protein, accompanied by ultrastructural defects in dynein arms and impaired ciliary motility. These findings align with previous reports and underscore the critical role of CFAP300 in dynein arm assembly and ciliary function [5,8]. The absence of ODA and IDA observed via TEM in our patient samples corroborates the known role of CFAP300 in dynein arm preassembly.

The pathogenic *CFAP300* variant c.198_200delinsCC results in severe ciliary dysfunction, as demonstrated by the significantly reduced CBF in both the ex vivo nasal epithelium (1.1 Hz) and ALI-cultured samples (1.5 Hz) compared to healthy controls (ex vivo: 5.8 Hz; ALI cultures: 7.7 Hz). Notably, the ALI cultures of patients and healthy donors showed higher CBF values than ex vivo samples. Our data exhibits a slight contradiction with previously published reports evaluating CBF ex vivo and in vitro in PCD patients with different genotypes. In the research conducted by Hirst et al., PCD ciliary samples exhibited a reduced mean CBF following ALI culture compared to native samples (from approximately 7.3 Hz to approximately 4.1 Hz post-culture) [17]. A study by Coles et al. showed a decrease in mean CBF after ALI culturing (ex vivo: 14.8 Hz; ALI cultures: 13.9 Hz; *p* = 0.03) in a group of patients with suspected PCD. At the same time, patients with equivocal PCD diagnosis showed increased CBF following ALI culturing (ex vivo: 13.9 Hz; ALI cultures: 14.4 Hz), while confirmed PCD cases exhibited complete ciliary immotility (0 Hz) in both native and cultured samples [14]. As shown, ALI culturing causes only slight changes in CBF while preserving the native sample’s phenotype, making ALI cultures a useful tool for evaluating cilia function in PCD.

Immunofluorescence analysis revealed a complete absence of the CFAP300 protein in patient-derived cells, while its expression was observed in wild-type controls, consistent with the protein colocalization staining analysis. This finding aligns with the emerging diagnostic practices in this field, as evidenced by the use of immunostaining panels in identifying the absence of dynein arm proteins in patients diagnosed with PCD [29,30,31]. Schultz et al. demonstrated by immunofluorescence analysis that ciliary samples from patients with the LoF mutation in *CFAP300* exhibited the complete absence of both IDA and ODA proteins (*DNAH7*, *DNAH5*, *DNAI1*), while control samples displayed normal protein localization patterns [5]. The absence of protein staining indicates that *CFAP300* gene mutations disrupt dynein arm assembly.

In conclusion, our results confirm the pathogenic impact of *CFAP300* mutations in PCD and emphasize the importance of integrating genetic, functional and immunofluorescence analyses for accurate diagnosis. Our study highlights the diagnostic utility of ALI cultures followed by ciliary analysis, which eliminates the confounding factors associated with native cilia from nasal epithelium biopsy samples. The consistency of our results with previous studies confirms the role of CFAP300 in dynein arm assembly and ciliary motility. Importantly, our findings demonstrate that CFAP300-associated PCD exhibits age-dependent progression, with older patients developing severe pulmonary complications. This underscores the need for early diagnosis and lifelong multidisciplinary management to mitigate disease progression and improve patient outcomes. Future work should extend this approach to other *CFAP* genes and dynein assembly factors to further elucidate the genotype–phenotype correlations in PCD, with validation in larger, multicenter cohorts.

## 4. Materials and Methods

### 4.1. Subject

This study was approved by the Ethics Committee of the Research Centre for Medical Genetics № 4 of 19.04.21 (Moscow, Russia) and conducted in accordance with the provisions of the Declaration of Helsinki of 1975. Samples were collected from three PCD patients and three healthy donors after obtaining written informed consent. Clinical data, PICADAR score, age, gender and respiratory microbiology were collected from electronic medical records, anonymized and transcribed. The PICADAR prediction tool is based on a 7-point scale, including (1) pre-term or full-term patient born; (2) neonatal respiratory symptoms requiring supplemental oxygen; (3) chronic wet cough beginning in infancy; (4) persistent perennial rhinitis; (5) recurrent otitis media in childhood; (6) organ laterality defects (including situs inversus or heterotaxy); and (7) congenital cardiac anomalies [1,32]. Table 4 presents the main characteristics of PCD patients and healthy donors.

### 4.2. Nasal Biopsies Collection

The nasal epithelium was obtained by brush biopsy. Brush biopsies were performed on each patient and healthy donors for video microscopy analysis ex vivo, TEM and nasal epithelial cell isolation. A disposable cytological brush BC-202D-5010 with a length of 1150 mm and a diameter of 5.0 mm (Olympus Medical Systems Corp., Tokyo, Japan) was used. Nasal epithelial biopsies were placed immediately after sampling into a buffer (0.9% NaCl solution) heated to 37 °C. Suitable research material was obtained from the inferior nasal meatus, which must contain intact epithelial layers with minimal contamination by erythrocytes and mucus.

### 4.3. Genetics

For the molecular genetic analysis, blood samples were collected from PCD patients. Genomic DNA was isolated using standardized extraction protocols, followed by whole-exome sequencing. Target enrichment was performed using the Illumina TruSeq^®^ Exome Kit (Illumina, San Diego, CA, USA) supplemented with custom-designed oligonucleotides (IDT xGen Exome Research Panel v1.0), covering the coding regions of more than 20,000 protein-coding genes. Paired-end sequencing (2 × 150 bp) was carried out on an Illumina NextSeq 500 platform. Sequencing data were processed using Illumina’s BaseSpace software (Enrichment v3.1.0, Illumina, San Diego, CA, USA). Genetic variants were filtered based on population frequency, retaining only those with a minor allele frequency below 1% in the Genome Aggregation Database (gnomAD v2.1.1). Focus was placed on coding variants, including missense, nonsense, small insertions/deletions (indels), and splice-site alterations. The pathogenicity of identified variants was evaluated in accordance with the American College of Medical Genetics and Genomics guidelines.

To validate the pathogenic variant, direct automated Sanger sequencing was performed [33]. Primers flanking the mutation were designed using the NCBI Primer-BLAST tool (https://www.ncbi.nlm.nih.gov/tools/primer-blast/, accessed on 10 June 2025), forward GACATCTGTACTTGGACACACA, reverse TTTACCTAATATGATCCATTGTCCA. The read length of the fragment was 294 nucleotides. Sequencing was performed on an ABI Prism 3130xl Genetic Analyzer (Applied Biosystems, Foster, CA, USA) according to the manufacturer’s protocol. Sequencing results were analyzed using Benchling website (Available online: www.benchling.com (accessed on 30 June 2025)).

### 4.4. Transmission Electron Microscopy

To evaluate ultrastructural alterations in the cilia of the nasal epithelium, brush biopsy specimens were fixed in 2.5% glutaraldehyde in 0.1 M cacodylate buffer for 24 h, followed by post-fixation in 1% osmium tetroxide in distilled water for 2 h. Samples were then dehydrated through a graded ethanol series (50% for 15 min, 70% for 12 h, and 96% in two 60 min steps) and subsequently in acetone (30 min). Following dehydration, tissue infiltration was performed using increasing concentrations of epoxy resin (Epon 812, DDSA, MNA, and DMP, prepared according to the manufacturer’s protocol) diluted in acetone (1:2 for 1 h, 1:1 for 1 h, and 2:1 for 12 h). The samples were then embedded in pure epoxy resin with an accelerator and polymerized at 37 °C for 24 h, followed by 60 °C for an additional 24 h.

Spermatological examination in men was carried out according to the recommendations of the WHO guidelines 2021 (Available online: https://www.who.int/publications/i/item/9789240030787 (accessed on 3 June 2025)). For the electron microscopy of spermatozoa, samples of native ejaculate after dilution were fixed with 2.5% glutaraldehyde solution on 0.1 M cacodilate buffer (pH 7.2), post-fixed with 1% osmic acid for 1 h and poured into Epon 812.

Ultrathin sections of the ciliated epithelium and spermatozoa were obtained on the ultramicrotome Reichert Jung ultramicrotome, Ultracut E (Reichert-Jung, Vienna, Austria), using a diamond knife (DiATOME Ultra 35deg Diamond Knife 1.5 mm, Sierre, Switzerland). The preparations were examined in a JEM-1011 transmission electron microscope (JEOL, Akishima, Japan) equipped with an Orius SC1000 W camera (Gatan Inc., Pleasanton, CA, USA) at an accelerating voltage of 80 kV. Ultrastructural studies were supported by the Moscow State University Development Program (PNR 5.13).

### 4.5. Culturing and Ciliogenesis of Nasal Epithelial Cells

The culturing and ciliogenesis of nasal epithelial cells were performed according to the previously published method [34]. In brief, nasal brush biopsies were placed in a sterile tube containing a transport medium consisting of DMEM (PanEco, Moscow, Russia), 10 µg/mL of fungin (InvivoGen, Toulouse, France), 100 u/mL of penicillin and 100 µg/mL of streptomycin (PanEco, Moscow, Russia), then centrifuged for 5 min at 1500× *g* and placed on a culture plastic pre-coated with Matrigel (Corning, NY, USA) in a medium for airway epithelial cells. The medium for airway epithelial cells consisted of PneumaCult™-Ex Plus Medium (StemCell Technologies, Vancouver, BC, Canada), 1 µM of A83-01 (Tocris, Bristol, UK), 1 µM of DMH1 (Tocris, Bristol, UK), 1 µM of hydrocortisone (Sigma Aldrich, Saint Louis, MO, USA), 50 u/mL penicillin and 50 µg/mL streptomycin (PanEco, Moscow, Russia). The nasal epithelial cells were passaged every 4–5 d using a 0.25% Trypsin solution (PanEco, Moscow, Russia).

The ciliogenesis of cultures of nasal epithelial cells was performed by ALI culturing. Nasal epithelial cells were passaged onto the inserts with a pore size of 0.4 μm and a diameter of 6.5 mm (StemCell Technologies, Vancouver, BC, Canada) at an amount of 30 × 10^3^ cells/insert in medium for airway epithelial cells. The cells were counted using an automatic Countess II cell counter (Thermo Fisher Scientific, Waltham, MA, USA). When the cells reached a monolayer, the medium above the insert was completely removed and the medium below the insert was replaced with ALI culture medium. The ALI culture medium consisted of PneumaCult™-ALI Medium (StemCell Technologies, Vancouver, BC, Canada), 4 μg/mL heparin (StemCell Technologies, Vancouver, BC, Canada), 9.6 μg/mL hydrocortisone (StemCell Technologies, Vancouver, BC, Canada), 50 units/mL penicillin and 50 μg/mL streptomycin. ALI culturing was performed for 24 d with medium changes every 48 h.

### 4.6. Video Microscopy Analysis

Biopsy specimens or ALI cultures were placed on glass slides in prewarmed DPBS. Video microscopy analysis was performed using a Vert A1 microscope (Zeiss, Suzhou, China) and a removable 1.5 MP ½.9 Sony Exmor CMOS Sensor (E3ISPM01500KPA, Sony, Tokyo, Japan) at ×100 and ×200 magnification and 90 frames per second (fps).

To assess CBF, video images were analyzed using the developed program “Program for determining the beating frequency of the ciliated epithelium in primary ciliary dyskinesia” (PCD High-Speed Video Microscopy Analysis (PCD HSVMA)), registration number No2023687245 [35]. CBP was determined by two independent expert operators.

### 4.7. Immunofluorescence Staining

To confirm the attainment of nasal epithelial cells and to confirm differentiation into ciliated cells, immunofluorescence staining for the markers of progenitor epithelial cells (Cytokeratin 5 and TP63) and the markers of ciliated cells (β-tubulin) was performed. Also, the ALI cultures of all PCD patients and HC1 were stained for the CFAP300 protein antibody to assess the presence and localization of the protein.

For this purpose, nasal epithelial cell cultures and ALI cultures were fixed in a cold 4% formalin solution for 15 min at room temperature (25 °C, RT). DPBS washings were performed twice. Cells were then permeabilized in 0.1% Tween 20 solution for 10 min at RT, washed three times with DPBS and blocked in 1% BSA and 0.1% Tween 20 for 30 min at RT. Thereafter, primary antibody solutions were added: for nasal epithelial cells, TP63 (Cat. No. 703809, RRID AB_2809251, Thermo Fisher Scientific, Waltham, MA, USA) was added at a concentration of 5 μg/mL and Cytokeratin 5 (Cat. No. ab52635, RRID AB_869890, Abcam, Cambridge, MA, USA) at 3 μg/mL; for ALI cultures, β-tubulin (Cat. No. ab131205, RRID AB_11156034, Abcam, USA) and CFAP300 (Cat. No. PAW426Hu01, Cusabio, Wuhan, China) were added. The cultures were incubated for 1 h at RT, followed by three washes with DPBS. Subsequently, a solution of secondary antibodies, namely Goat Anti-Mouse IgG H&L (Alexa Fluor^®^ 488) (RRID AB_2630356, Abcam, Cambridge, MA, USA) and Goat Anti-Rabbit IgG H&L (Alexa Fluor^®^ 594) (RRID AB_2650602, Abcam, Cambridge, MA, USA), was added at a concentration of 10 μg/mL and incubated for 1 h at RT. Cells were washed three times with DPBS. DAPI (Abcam, Cambridge, MA, USA) was added and incubated for 10 min at RT. The visualization of stained nasal epithelial cells was performed on an automated Lionheart FX imager (BioTek, Layton, UT, USA). For the visualization of ALI cultures, stained inserts were placed in a solution of 2.5 mM of fructose in 60% glycerol and incubated for 20 min at RT. Then, the solution was transferred to a slide, a coverslip was placed on top and microscopy was performed on a Leica TCS SP8 confocal laser scanning microscope (Leica Microsystems, Wetzlar, Germany).

To assess the colocalization of CFAP300 and β-tubulin staining, five image fields were acquired for each of the six ALI culture samples (PCD1-3, HC1-3). Colocalization analysis was performed using the MeasureColocalization module in CellProfiler software v4.2.4 (Broad Institute of MIT and Harvard, Cambridge, MA, USA). The evaluation was conducted across entire images on a pixel-by-pixel basis. Quantitative assessment was performed using Manders’ coefficient, which determines the fraction of the CFAP300-positive signal colocalized with β-tubulin. This was calculated as the ratio of the sum of red channel intensities (CFAP300) in pixels containing a green signal (β-tubulin) to the total sum of red channel intensities. Additionally, the rank-weighted colocalization coefficient (RWC) was applied, which evaluates the quality of CFAP300/β-tubulin colocalization while accounting for intensity differences between channels. The weighting factor was determined as a function of the rank difference in intensity between channels. Threshold values for analysis were set as a percentage of the maximum image intensity.

### 4.8. Statistical Analysis

Statistical analysis was performed in GraphPad Prism v8 (GraphPad Software Inc., San Diego CA, USA). Quantitative data were summarized using the median and interquartile range (Q1–Q3). To assess differences in CBF between the PCD and HC groups and between ex vivo and in vitro ciliary cultures, we used a nested *t*-test. The statistical analysis of protein colocalization data (CFAP300 and β-tubulin) was performed using unpaired Student’s *t*-test. Differences were considered significant at *p* < 0.05.

## Figures and Tables

**Table 1:** Clinical and anamnestic data of subjects.

Parameter	PCD1	PCD2	PCD3
Neonatal period	Unremarkable	Intrauterine hydrocephalus, congenital pneumonia, admitted to NICU on day 1	Congenital pneumonia
Family history of PCD or situs inversus	−	+(grandfather with Kartagener syndrome)	−
Situs inversus (Kartagener syndrome)	−	+	+
Bronchiectasis	+	+	+
PICADAR score	8	12	12
FEV1 (% predicted, normal ≥80%)	99.9%	66%	44%
Pseudomonas aeruginosa	−	+	+
Chronic rhinosinusitis	+	+	+
Nasal polyposis	−	−	+
Otitis	−	+	+
Hearing loss severity	−	Mild (grade 1)	Moderate (grade 1–2)
IV anti-pseudomonal therapy (per year)	−	2–3 courses	1–2 courses
Inhaled anti-pseudomonal therapy	−	Tobramycin (continuous)	Colistimethate sodium (continuous)
Oral antibiotics for exacerbations (per year)	1–2 courses	4–5 courses	4–6 courses

Abbreviations: FEV1—forced expiratory volume in 1 s.

**Table 2:** Results of ex vivo video microscopy analysis, molecular genetics and TEM analysis of PCD patients.

Patients with PCD	Genotype	TEM	CBP Ex Vivo	CBF Ex Vivo (Median (Q1–Q3)), Hz	*p*-Value (vs. HC)
PCD1	CFAP300 c.198_200delinsCC homo/hemizygous	Complete lack of ODA and IDA	Immotile cilia	1.25 (0.3–1.9)	*p* < 0.0001
PCD2			N/A	N/A	N/A
PCD3			Immotile cilia	0.9 (0.5–1.0)	*p* < 0.0001

Abbreviations: TEM—transmission electron microscopy, ODA—outer dynein arm, IDA—inner dynein arm, CBP—cilia beating pattern, N/A—not analyzed, CBF—cilia beating frequency, vs.—versus.

**Table 3:** Results of video microscopy analysis of ALI-cultured ciliated epithelium.

	CBP In Vitro	CBF In Vitro (Median (Q1–Q3)), Hz	*p*-Value (vs. HC)
Patients with PCD			
PCD1	Almost immotile cilia, only minimal residual ciliary movements	1.5 (1.4–1.8)	*p* = 0.0008
PCD2	Immotile cilia	1.3 (1.1–1.6)	
PCD3	Immotile cilia	1.6 (0.5–3.1)	
Healthy donors			
HC1	Regular ciliary beating	8.0 (7.7–8.9)	
HC2		6.6 (5.6–7)	
HC3		6.9 (6.3–7.2)	

Abbreviations: CBP—cilia beating pattern, CBF—cilia beating frequency, vs.—versus.

**Table 4:** Baseline characteristics of PCD patients and healthy donors.

Patients	Gender	Age
Patients with PCD		
PCD1	F	23
PCD2	M	28
PCD3	F	35
Healthy donors		
HC1	F	31
HC2	M	26
HC3	F	25

Abbreviations: F—female; M—male.

## Data Availability

The data presented in this study are available upon request from the corresponding author.

## Supplementary Materials

The following supporting information can be downloaded at: https://www.mdpi.com/article/10.3390/ijms26157655/s1.

## Abbreviations

The following abbreviations are used in this manuscript:

PCD	Primary Ciliary Dyskinesia
CFAP300	Cilia and Flagella-Associated Protein 300
ALI	Air–Liquid Interface
ODA	Outer Dynein Arm
IDA	Inner Dynein Arm
TEM	Transmission Electron Microscopy
CBF	Ciliary Beat Frequency
CBP	Ciliary Beat Pattern
SCD	Secondary Ciliary Dyskinesia
NICU	Neonatal Intensive Care Unit
FEV1	Forced Expiratory Volume in 1 s
IVF	In Vitro Fertilization
N/A	Not Analyzed
RT	Room Temperature
fps	Frames Per Second
