# Supplementary material for: CFAP300 Loss-of-Function Mutations with Primary Ciliary Dyskinesia: Evidence from Ex Vivo and ALI Cultures
Source: Int J Mol Sci. 2025 Aug 7;26(15):7655. doi: 10.3390/ijms26157655 (PMC12347484; doi:10.3390/ijms26157655)
Supplement: Supplementary file 1 [file ijms-26-07655-s001.zip › ijms-3789049-supplementary.pdf]

Supplementary File

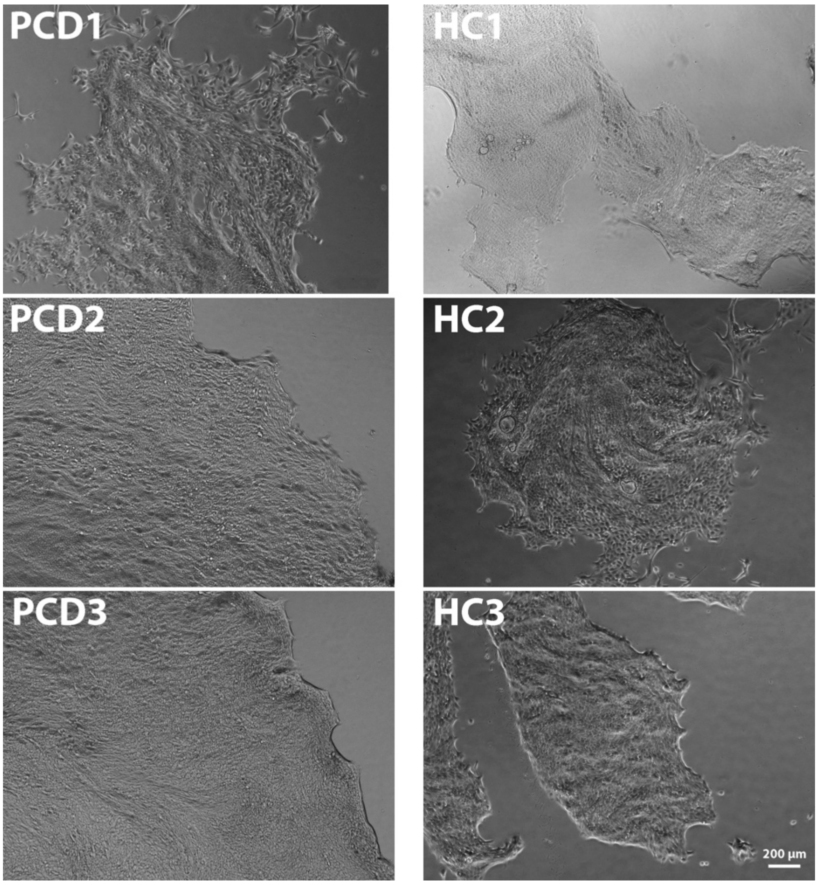

Supplementary Figure S1. Phase-contrast images of nasal epithelial cells from six donors at passage 0. Scale bar 200 μm
